# Cytoprotective Effects of Fish Protein Hydrolysates against H_2_O_2_-Induced Oxidative Stress and Mycotoxins in Caco-2/TC7 Cells

**DOI:** 10.3390/antiox10060975

**Published:** 2021-06-18

**Authors:** Mercedes Taroncher, Yelko Rodríguez-Carrasco, Tone Aspevik, Katerina Kousoulaki, Francisco J. Barba, María-José Ruiz

**Affiliations:** 1 Department of Preventive Medicine and Public Health, Food Science, Toxicology and Forensic Medicine, Faculty of Pharmacy, University of Valencia, 46100 Burjassot, Valencia, Spain; mercedes.taroncher@uv.es (M.T.); yelko.rodriguez@uv.es (Y.R.-C.); M.jose.ruiz@uv.es (M.-J.R.); 2 Department of Nutrition and Feed Technology, Nofima AS, 5141 Bergen, Norway; tone.aspevik@Nofima.no (T.A.); katerina.Kousoulaki@Nofima.no (K.K.)

**Keywords:** fish hydrolysates, cytotoxicity, oxidative stress, cytoprotective effect, bioavailability

## Abstract

Many studies report the potent antioxidant capacity for fish protein hydrolysates, including radical scavenging activity and inhibition ability on lipid peroxidation (LPO). In this study, the in vitro cytotoxicity of protein hydrolysates from different salmon, mackerel, and herring side streams fractions was evaluated in the concentration range from 1 to 1:32 dilution, using cloned human colon adenocarcinoma cells TC7 (Caco-2/TC7) by MTT and PT assays. The protein hydrolysates’ antioxidant capacity and oxidative stress effects were evaluated by LPO and reactive oxygen species (ROS) generation, respectively. The antioxidant capacity for pure and bioavailable hydrolysate fraction was also evaluated and compared. Additionally, mycotoxin levels were determined in the fish protein hydrolysates, and their cytoprotective effect against T-2 toxin was evaluated. Both hydrolysates and their bioavailable fraction induced similar cell viability rates. The highest cytoprotective effect was obtained for the salmon viscera protein hydrolysate (HSV), which increased the cell viability by 51.2%. ROS accumulation induced by H_2_O_2_ and LPO was suppressed by all pure hydrolysates. The cytoprotective effect of hydrolysates was observed against T-2. Moreover, the different fish fraction protein hydrolysates contain variable nutrients and unique bioactive peptide composition showing variable bioactivity, which could be a useful tool in developing dietary supplements with different target functional properties.

## 1. Introduction

Synthetic antioxidants are commonly used to preserve food products, including butylated hydroxytoluene (BHT), tert-butyl hydroquinone (TBHQ) and butylated hydroxyanisole (BHA), but their use is limited due to the potential toxic effects in humans [1,2,3]. Therefore, there is an increasing consumer demand for natural antioxidants in order to delay food deterioration caused by oxidation, as well as to alleviate the oxidative stress caused by mitochondrial generation of reactive oxygen species (ROS) [4,5,6,7], proteins and peptides being of great interest [8,9,10]. 

Protein hydrolysates and peptides exhibit multiple physiological functionalities such as antimicrobial, antioxidant, antihypertensive, and cholesterol-lowering effects and immunomodulatory activities [11,12,13]. Moreover, in some recent studies, the ability of fish protein hydrolysates to suppress DSS-Induced colitis by modulating intestinal inflammation in mice, as well as to suppresses diabetes and modulates intestinal microbiome in a murine model of diet-induced obesity, has been observed [14,15]. 

Filleting fish generates large amounts of protein-rich side stream materials, such as heads, backbones, viscera, and trimmings, with potential use for human consumption when handled properly and according to food-grade regulations. In order to use side stream fish proteins directly, the production of fish protein hydrolysates is a promising option, where the proteins are cleaved into smaller and more water-soluble peptides compared with the native protein [16]. 

Fish protein hydrolysates can have great application potential in the food, medical, and cosmetic industries [17]. An example is collagen, abundant in connective tissue rich fish fractions, such as skin and bones [18]. Marine collagen has shown promising activities such as antioxidant, wound healing, anti-aging, the adipogenic differentiation inhibition, and anti-freezing, among others [13,19,20,21,22]. Numerous studies have reported that peptides derived from fish collagen and protein hydrolysates, of a variety of fish species such as tuna, salmon, cuttlefish, sardinella, tilapia, and shrimp, showed potent antioxidant activities including radical scavenging activity, reducing power, and inhibition ability on lipid and protein oxidation [23,24]. Moreover, Zhao et al. [25] and Wong et al. [26] reported that protein hydrolysates of miiuy croaker swim bladders and hydrolysate of blue-spotted stingrays could scavenge radicals and inhibit lipid peroxidation. Yarnpakdee et al. [27] studied lipid oxidation in protein hydrolysates from the muscles of Indian mackerel. Sheriff et al. [28] reported powerful antioxidant, lipid peroxidation inhibition and free radical scavenging activity of hydrolysate from the backbones of Indian mackerel. Kumar et al. [29] found a good ability to scavenge hydroxyl radicals from antioxidant peptides obtained from horse mackerel viscera protein. 

The bioavailability of fish protein hydrolysates has been evaluated in several studies [30,31,32,33,34] using the in vitro digestion technique. The human colorectal adenocarcinoma (Caco-2) cell line is the international validated model used for evaluating the intestinal absorption of food nutrients, also recognized as a reliable cell model for cell-based bioassays of food antioxidant activity [35]. These cells are able to differentiate in long-term culture and to polarize, when seeded on semi-permeable membranes, on which they form a continuous monolayer with tight junctions mimicking the intestinal barrier [36]. Nevertheless, Caco-2 cells are heterogeneous, and their physiological performance is highly dependent on culture conditions, often leading to variable transport properties and permeability. In order to reduce the heterogeneity of the cells, several clones have been isolated from Caco-2 cells. The TC7 clone was obtained from a late passage of the parental Caco-2 cell line and characterized for its ability to transport [36]. Zucco et al. [37] compared the characteristics of four Caco-2 cell lines and concluded that the TC7 clone consisted of a more homogeneous cell population, with more representative functions of the small intestinal enterocytes. Thus, in our study we evaluated the suitability of Caco-2/TC7 as a predictive in vitro model for the intestinal transport of fish protein hydrolysate compounds.

Mycotoxins are secondary toxic metabolites produced by fungi found in cereals and their side streams, monitored in both food and feed by national and international public health programs to evaluate compliance with the current regulations [38,39]. Aquaculture fish are commonly exposed to feed borne mycotoxins, especially from wheat and maize derived raw materials largely used in aquafeed formulations due to their favorable availability, price, and protein content [40]. There are severable studies evaluating the presence of mycotoxins in terrestrial animals, whereas the data on the occurrence of mycotoxins in farmed fish is limited [41].

The aims of this study were: (i) to evaluate the viability of Caco-2/TC7 cells after 24 h exposure of protein hydrolysates based on heads, backbones, and viscera from salmon, mackerel, and herring (the latter providing only viscera), and fish collagen based on flounder skin before and after an in vitro digestion process; (ii) to determine the cytoprotective/cytotoxic effects of these protein hydrolysates against oxidative stress-induction in Caco-2/TC7 cells; (iii) to identify possible fungal metabolites in these hydrolysates using a liquid chromatography high resolution mass spectrometry technique, and to evaluate the cytoprotective effect of hydrolysates in co-exposure with T-2 toxin.

## 2. Materials and Methods

### 2.1. Reagents

The reagent grade chemicals and cell culture compounds used, namely Dulbecco’s Modified Eagle’s Medium (DMEM + GlutaMAX™), antibiotic solution (penicillin-streptomycin), non-essential amino acids (NEAA), 4-(2-hydroxyethyl)-1-piperazineethanesulfonic acid (HEPES), fungizone, trypsin/EDTA solutions, Phosphate Buffer Saline (PBS), Fetal Bovine Serum (FBS), methylthiazoltetrazolium salt (MTT) dye, dimethyl sulfoxide (DMSO), thiobarbituric acid (TBA), deferoxamine mesylate salt (DFA), di-ter-butylmethylphenol (BHT), 1,3,3,3 tetramethoxipropan (TMP), 2′,7′-dichlorodihydrofluorescein diacetate (H_2_-DCFDA), t-octylphenoxypolyethoxyethanol, glacial acetic acid, H_2_O_2_, NaOH, NaCl, glycine, Coomassie Brilliant Blue, ethyl acetate, and CaCl_2_, were purchased from Sigma-Aldrich (St. Louis, MO, USA). Methanol (MeOH) was purchased from VWR International (LLC, Monroeville, PA, USA). Deionized water (resistivity < 18 MΩ cm) was obtained by filtering tap water through a Milli-Q water purification system (Millipore, Bedford, MA, USA). Standards of mycotoxins were purchased from Sigma-Aldrich (St. Louis, MO, USA).

Enzymatic protein hydrolysates based on mackerel heads (HMH), mackerel backbones (HMB), mackerel viscera (HMV), salmon heads (HSH), salmon backbones (HSB), salmon viscera (HSV), and herring viscera (HHV) were produced according to Aspevik et al. [42]. In brief, the raw materials were mixed with tap water (1:1) and subjected to enzymatic hydrolysis for 50 min at 55 °C using the protease Food Pro PNL (DuPont Wilmington, DE). After thermal inactivation of the enzyme (>90 °C, 10 min), the water phase (hydrolysate) was separated from the insoluble fraction and lipids and further purified using a 0.1 µm ceramic membrane filter before spray drying to a light-yellow powder. The chemical properties of protein hydrolysates of mackerel and salmon are shown in Aspevik et al. [42]. Flounder skin collagen was kindly provided by Seagarden (Karmøy, Norway). 

### 2.2. Cell Culture and Treatment

The Caco-2/TC7 cells were cultured in DMEM medium, supplemented with 10% FBS, 1% HEPES, 1% NEAA, 0.2% fungizone, 100 U/mL penicillin, and 100 mg/mL streptomycin. The incubation conditions were pH 7.4, 5% CO_2_ at 37 °C and 95% air atmosphere at constant humidity. The medium was changed every 2–3 days. All experiments were carried out 7 days post-seeding, when the integrity of the Caco-2/TC7 cell monolayers had a measure of transepithelial electrical resistance (TEER) above 300 Ω/cm^2^. The clonal line Caco-2/TC7 cells were kindly provided by Central Service for Experimental Research (SCSIE) of the University of Valencia (Valencia, Spain).

### 2.3. In Vitro Cytotoxicity

Cytotoxic effects were determined in Caco-2/TC7 cells by the MTT and total protein content (PC) assays. The two assays have been extensively used for in vitro toxicological studies used to measure cell proliferation and survival. The MTT assay determines the viability of cells by the reduction in yellow soluble MTT, only in the metabolically active cells, via a mitochondrial-dependent reaction to an insoluble purple formazan crystal. The MTT viability assay was performed according to Ruiz et al. [43].

Briefly, the Caco-2/TC7 cells were plated in 96-well tissue culture plates at a density of 2 × 10^4^ cells/well. In Caco-2/TC7, the culture medium was replaced with fresh medium containing serial dilutions of each hydrolysate. The amount of 1 mg of the powdered hydrolysates was resuspended in 1 mL of fresh medium. Following this, 1:2 dilutions were carried out using fresh medium. The range of concentrations of the hydrolysates were: 1 (mg/mL), 1:2 (0.5 mg/mL), 1:4 (0.25 mg/mL), 1:8 (0.125 mg/mL), 1:16 (0.0625 mg/mL), and 1:32 (0.03125 mg/mL). The hydrolysates were exposed over 24 h. During the exposure time, neither the medium nor the hydrolysate was replenished. After 24 h of exposure, the medium was removed and 200 μL of fresh medium was added to each well. Then, 50 μL/well of MTT was added, and the plates were returned to the incubator in the dark. After 3 h of incubation, the MTT solution was removed and 200 μL of DMSO was added, followed by 25 μL of Sorensen’s glycine buffer. Plates were gently shaken for 5 min to achieve the complete dissolution. The absorbance was measured at 540 nm using an automatic ELISA plate reader (MultiSkanEX, Thermo Scientific, Walthman, MA, USA).

The PC method is based on the increase in absorbance of Coomassie Brilliant Blue dye when binding to proteins. The assay was performed in the same 96-well plates where the MTT test was carry out. First, the plates were washed with PBS and each well received 200 μL of NaOH 0.1 N to dissolve the proteins. After 2 h of incubation, 170 μL of NaOH was removed from each well and 180 μL of diluted 22% Coomassie Brilliant Blue was added. The plates remained for 30 min at room temperature and the absorbance was measured at 620 nm in an automatic ELISA plate reader (MultiSkanEX, Thermo Scientific, Walthman, MA, USA). 

For MTT and PC assays, cell viability was expressed as a percentage relative to the control solvent (medium DMEM + GlutaMAX™). Determinations were performed in three independent experiments with 4 replicates for each one. The mean inhibition concentration (IC_50_) values were calculated using SigmaPlot version 11 (Systat Software Inc., GmbH, Düsseldorf, Germany).

### 2.4. Intracellular ROS Generation

Early intracellular ROS production was monitored in Caco-2/TC7 cells by adding H_2_-DCFDA. The H_2_-DCFDA is taken up by cells and then deacetylated by intracellular esterase’s; the resulting non-fluorescent 2′,7′-dichlorodihydrofluorescein (H_2_-DCF) is switched to highly fluorescent dichlorofluorescein (DCF) when oxidized by ROS. The generation of ROS was monitored according to Ruiz-Leal and George [44]. Briefly, 2 × 10^4^ cells/well were seeded in a 96-well black culture microplate. In Caco-2/TC7 cells the culture medium was replaced, and cells were loaded with 20 μM H_2_-DCFDA in a fresh medium for 20 min. Then two assays (A and B) were simultaneously carried out. Assay A: H_2_-DCFDA was removed and 200 μL/well of fresh medium or medium with hydrolysates 1 mg/mL was added. Assay B: H_2_-DCFDA was removed and 200 μL/well of fresh medium or medium with hydrolysates (1 mg/mL) and 5 mM H_2_O_2_ was added. In both assays, the ROS level was monitored by measuring the increase in fluorescence on a Wallace Victor^2^, model 1420 multilabel counter (PerkinElmer, Turku, Finland), at intervals up to 2 h at excitation/emission wavelengths of 485/535 nm, respectively. Results were expressed as fluorescence intensity. Determinations were performed in three independent experiments with 16 replicates each.

### 2.5. Lipid Peroxidation Assay

Lipid peroxidation (LPO) assay was carried out by determining the formation of reactive thiobarbituric acid reactive substances (TBARS), according to Ferrer et al. [45]. The TBARS allow us to determine the production of a red adduct between TBA and malondialdehyde (MDA), which is a biomarker used to prove that the LPO process has occurred. Briefly, 2.25 × 10^5^ cells/well were seeded in six-well plates. In Caco-2/TC7 cells two assays (A and B) were simultaneously carried out after confluence. Assay A: the culture medium was replaced with fresh medium with hydrolysates 1 mg/mL. Assay B: the culture medium was replaced with fresh medium with 5 mM H_2_O_2_ for 5 min, except the control. After that, the culture medium with H_2_O_2_ was replaced with fresh medium with hydrolysates (1 mg/mL). In both assays, the Caco-2/TC7 cells were exposed to 1 mg/mL of hydrolysates for 24 h. Then, the medium was removed, and cells were washed with PBS, homogenized in 150 mM sodium phosphate buffer (NaH_2_PO_4_) pH 7.4 and lysate with the Ultra-Turrax T8 IKA^®^-WERKE. Immediately, cells were boiled at 100 °C in a water bath for 20 min under acidic conditions in the presence of 0.5% TBA, 1.5 mM DFA, and 3.75% BHT. After that, the samples were placed on ice for 5 min and centrifuged at 2880× *g* for 15 min. The absorbance was measured at 532 nm. Three independent experiments were conducted with three replicates each. Results were expressed as ng of MDA/mg of protein measured by the Lowry method.

### 2.6. Comparison of Cytotoxicity between Pure Hydrolysates and Their Bioavailable Fraction

The Caco-2/TC7 were exposed to 0.0625 mg/mL of bioaccessible fraction of each hydrolysate. To obtain the bioaccessible fraction, the standardized method INFOGEST was applied. Simulated Salivary Fluid (SSF), Simulated Gastric Fluid (SGF), Simulated Intestinal Fluid (SIF), and enzymatic activity assays were prepared according to Minekus et al. [46]. Due to the high percentage of proteins in the hydrolysate samples, the salivary step was carried out without amylase enzyme. Briefly, 2.5 mL of hydrolysate solution, 2 mL of SSF, 12.5 μL of 0.3 M CaCl_2_, and deionized water to a final volume of 5 mL were mixed for 2 min. Afterwards, to simulate the gastric phase, 3.75 mL of SGF, 0.8 mL of pepsin solution (25.000 U/mL), and 2.5 μL of 0.3 M CaCl_2_ were added. Then, the pH was adjusted to 3.0, and deionized water was added up to a final volume of 10 mL. This gastric mixture was incubated for 2 h. Subsequently, 5.5 mL of SIF containing pancreatin (100 U trypsin activity/mL) and bile salts (10 mmol/L), and 20 μL of 0.3 M CaCl_2_ were added. The pH was adjusted to 7.0, deionized water was added up to a final volume of 20 mL, and the intestinal mixture was incubated for 2 h. The oral, gastric, and intestinal steps were performed by mechanical shaking at 95 *x*rpm and 37 °C. At the end of the in vitro digestive process, samples were cooled in an ice bath and centrifuged at 3100× *g* and 4 °C for 60 min to obtain the bioaccessible fraction.

**Figure 1:**
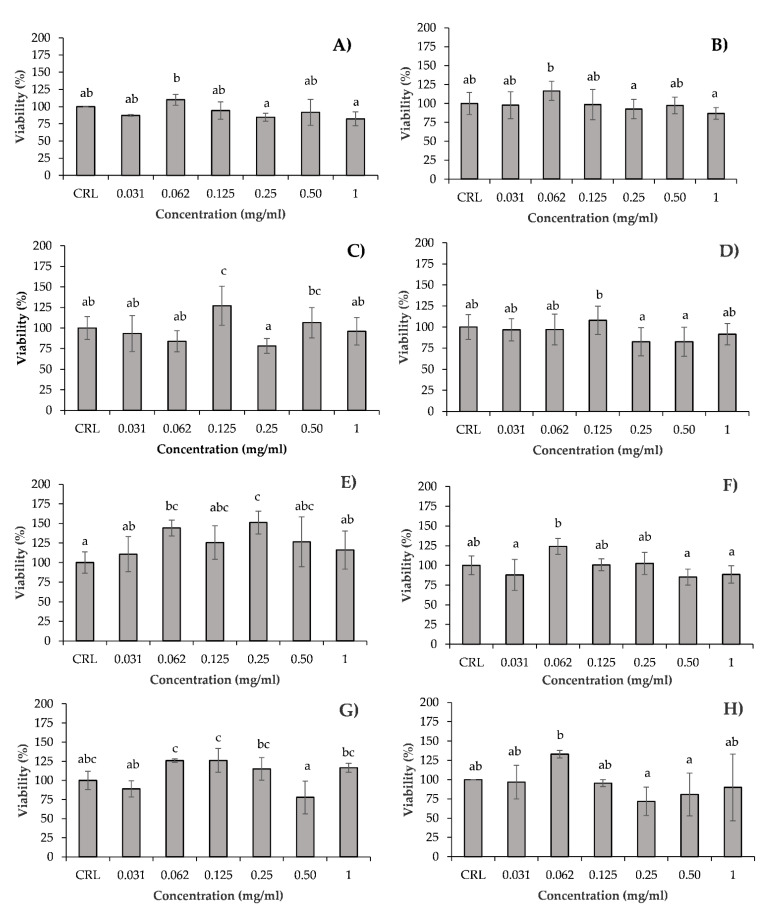
Effect of HSH (**A**); HMH (**B**); HSB (**C**); HMB (**D**); HSV (**E**); HMV (**F**); HHV (**G**); and collagen (**H**) on cell viability (MTT assay) in Caco-2/TC7 cells after 24 h of exposure at increasing concentrations from 0 to 1 mg/mL. Values are expressed as mean ± SEM (*n* = 3). Values in the same figure with different superscript letter are significantly different (*p <* 0.05). CRL: control.

The Caco-2/TC7 cells were seeded at a density of 2.25 × 10^5^ cells in inserts 6-well Transwell Permeable Supports, 12 mm diameter (Corning Life sciences, Corning, NY, USA) and 0.4 mm of pore size, and grown for 7 days. The culture medium in the apical and basolateral sides was replaced every 2–3 days. The integrity of the Caco-2/TC7 cell monolayers was confirmed by measuring the transepithelial electrical resistance (TEER) using an electric resistance device (Millicell-ERS, Millipore Corp), and monolayers with TEER above 300 Ω/cm^2^ were used for the bioavailability assay. Medium of apical (upper compartment) and basolateral side (lower compartment) was removed, and transport was assessed by paracellular passage of bioaccessible fraction of hydrolysates (0.0625 mg/mL) in the apical side to the basolateral side. Therefore, 1.5 mL of bioaccessible fraction of digested hydrolysates was added to the apical side and 2 mL of medium without serum was added to the basolateral side. Control samples composed by transport medium without serum were also evaluated. The medium of the basolateral side, which is the bioavailable fraction, is collected. Then, with the bioavailable fraction, the MTT assay was carried out to determine the cytotoxicity of bioavailable fraction of hydrolysates at 0.0625 mg/mL. The results obtained were compared with the pure hydrolysates at 0.0625 mg/mL. The concentration 0.0625 mg/mL was chosen as it is amongst those which exerted the highest cytoprotective effect in Caco-2/TC7 cells during the MTT assay (Figure 1). Determinations were performed in three independent experiments with 4 replicates each.

### 2.7. Cytoprotective Effect of Hydrolysates Exposed Together with T-2 Toxin

The T-2 toxin (T-2) is a *Fusarium* mycotoxin frequently occurring in cereals worldwide and the most toxic fusariotoxin. The recent animal dietary exposure to T-2 conducted by EFSA, reported the occurrence of T-2 in feed in concentrations of up to 405 μg/kg [47]. Hence, the cytoprotective effect of hydrolysates in Caco-2/TC7 cells was evaluated by MTT assay, exposing 60 nM T-2 to each hydrolysate at 0.0625 mg/mL. To develop the MTT assay, 2 × 10^4^ cells/well were plated in 96-well tissue culture plate and the assay was performed as described in Section 2.3 In vitro cytotoxicity. The results obtained were compared with cells exposed to 60 nM T-2 and pure hydrolysates at 0.0625 mg/mL individually. The concentration of 60 nM was chosen as it showed a cytotoxic effect in previous studies in our laboratory [48]. Determinations were performed in three independent experiments with 4 replicates each.

### 2.8. Determination of Fungal Metabolites in Fish Hydrolysates through Liquid Chromatography Quadrupole Time of Fliht Mass Spectrometry

The extraction of mycotoxins from hydrolysates of fish samples was performed according to the sample preparation procedure described by Taroncher et al. [48] slightly modified. In brief, 0.5 mL of hydrolysate fish sample was collected and transferred to a 4 mL Eppendorf Safe-Lock microcentrifuge tube. Then, 3 mL of ethyl acetate was added, and the mixture was shaken for 2 min with an Ultra-Turrax Ika T18 basic (Staufen, Germany) and centrifuged at 5600× *g* for 3 min (Centrifuge 5910R, Eppendorf, Germany). The supernatant phase was collected and evaporated under a gentle N_2_ stream at 45 °C to dryness with a TurboVap-LV (Zymark, Allschwil, Switzerland), re-dissolved in 0.14 mL of a mixture methanol:water (70:30, *v*/*v*), and then filtered through a 0.2 μm filter prior to the analysis.

The analysis was performed using a liquid chromatography quadrupole time of flight mass spectrometry (LC-Q-TOF MS) system consisting of an LC Agilent 1200-LC system (Agilent Technologies, Palo Alto, CA, USA) equipped with a vacuum degasser, an autosampler and a binary pump. The column was a Gemini NX-C_18_ (150 × 2 mm, i.d. 3 μm, Phenomenex, Torrance, CA, USA) with a guard column C_18_ (4 × 2 mm, i.d. 3 μm). Mobile phases consisted of Milli-Q water with 0.1% formic acid and 5 nM ammonium formate as solvent system A, and methanol with 0.1% formic acid and 5 nM ammonium formate as solvent system B, with the following elution gradient: 0.2 min, 20% B; 0.5 min, 20–40% B; 5 min, 100% B; and 2 min, decrease to 20% B, maintained for 6 min. The flow rate used was 0.200 mL/min, and the total run time was 14 min. The sample volume injected was 20 μL.

The mass spectrometry (MS) analysis was carried out using a 6540 Agilent Ultra-High Definition Accurate-Mass Q-TOF MS, equipped with an Agilent Dual jet Stream electrospray ionization (Dual AJS ESI) interface in both positive and negative ionization modes. The conditions were as follows: sheath gas temperature 350 °C at a flow rate of 8 L/min, capillary voltage 3500 V, nebulizer pressure 45 psig, drying gas 10 L/min, gas temperature 300 °C, skimmer voltage 65 V, octopole RF peak 750 V, and fragmentor voltage 130 V. Analyses were performed using AutoMS/MS mode in a mass range of 50–1200 *m*/*z*. The acquisition rate was 3 scans/s for three different collision energies (10, 20, and 40 eV). Internal mass correction was enabled using two reference masses in positive mode (121.050873 and 922.009798 *m*/*z*) and two reference masses in negative mode (112.985587 and 1033.988109 *m*/*z*). Instrument control and data acquisition were performed using Agilent MassHunter Workstation software B.08.00. Potential analytes were identified by the MassHunter METLIN Metabolite PCD (Personal Compound Database) and PCDL (Personal Compound Database and Library) from Agilent Technologies. 

### 2.9. Statistical Analysis

The statistical analysis of the data was carried out using the Statgraphics version 16.01.03 statistical package (IBM Corp., Armonk, NY, USA). Data were expressed as the mean ± standard error of the mean (SEM) of different independent experiments. The statistical analysis of the results was performed using the Student t-test for paired samples. Differences between groups were analyzed using the one-way analysis of variance (ANOVA), followed by a Tukey HDS post-hoc test for multiple comparisons. Statistical significance was considered as *p* *≤* 0.05 and tendencies as 0.1 *< p <* 0.05.

## 3. Results

### 3.1. In Vitro Cytotoxicity

The safe supplementation level of hydrolysates HSV, HSB, HSH, HMH, HMB, HMV, HHV, and collagen based on the evaluation of cytotoxic effect in Caco-2/TC7 cells was evaluated by MTT and PC assays over 24 h. The concentration range of the hydrolysates selected to study the potential cytotoxic/cytoprotective effects on these cells was: 1, 0.5, 0.25, 0.125, 0.0625, and 0.03125 mg/mL.

After 24 h of treatment, most hydrolysates did not show significant effects on the mitochondrial function of Caco-2/TC7 cells at any of the concentrations tested, except HSB at 0.125 mg/mL, and HSV at 0.0625 mg/mL and 0.25 mg/mL (Figure 1). The maximum measured MTT increase was 27% for HSB, and 51.2% for HSV. The increase in cell viability can be due to the hormetic effect of the test hydrolysates in Caco-2/TC7 cells.

**Figure 2:**
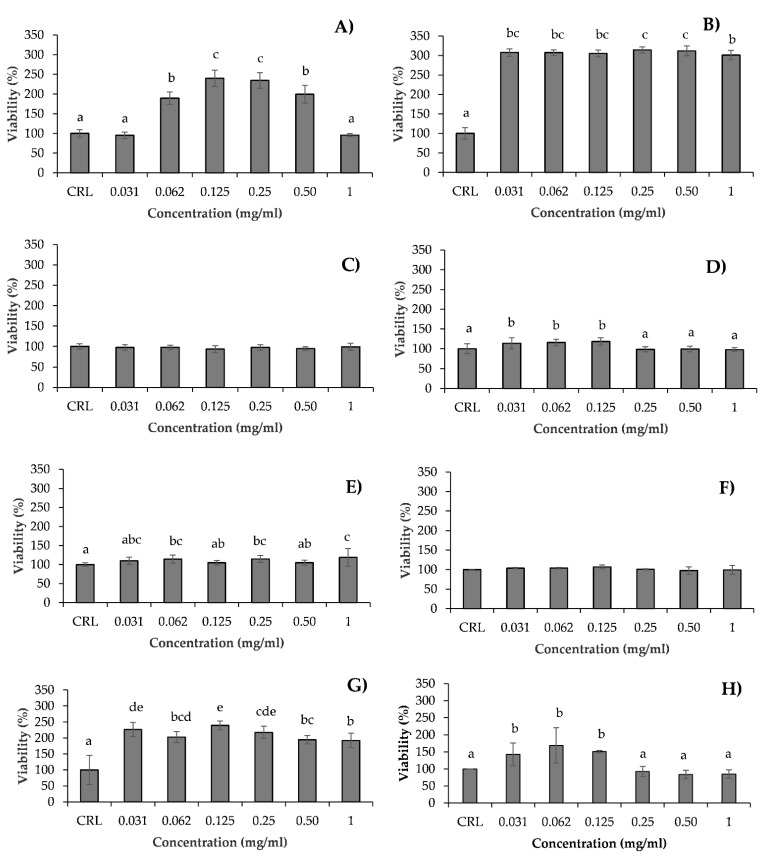
Effect of HSH (**A**); HMH (**B**); HSB (**C**); HMB (**D**); HSV (**E**); HMV (**F**); HHV (**G**); and collagen (**H**) on cell viability (PC assay) in Caco-2/TC7 cells after 24 h of exposure at increasing concentrations from 0 to 1 mg/mL. Values are expressed as the mean ± SEM (*n* = 3). Values in the same figure with different superscript letter are significantly different (*p <* 0.05). CRL: control.

In the PC assay (Figure 2) all hydrolysates, except HSB and HMV, showed a hormetic effect when the cells were exposed to specific dilutions, including 0.0625 mg/mL. The maximum measured PC increases were 18% for HMB, 19% for HSV, 69% for collagen, 139% for HHV, 140% for HSH, and 214% for HMH. At 0.0625 mg/mL used in the subsequent tests, the PC increasing effect of the different hydrolysates was as follows: HSV = HMB < collagen < HSH < HHV < HMH (*p =* 0.000).

### 3.2. Intracellular ROS Generation

**Figure 3:**
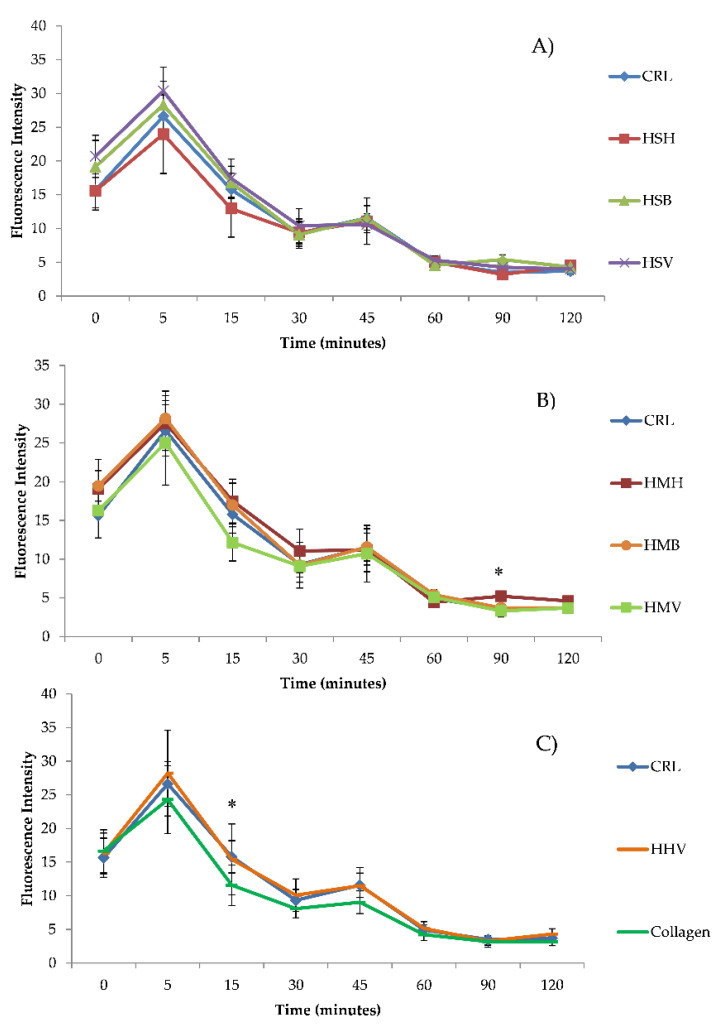
Time dependence of ROS-induced fluorescence in Caco-2/TC7 cells exposed to (**A**) salmon hydrolysates, (**B**) mackerel hydrolysates, and (**C**) herring hydrolysates and fish collagen at 1 mg/mL without oxidative stress induced by H_2_O_2_. Results are expressed as the mean ± SEM (*n* = 3). (*) *p* *≤* 0.05 indicates significant differences compared to the control (CRL).

**Figure 4:**
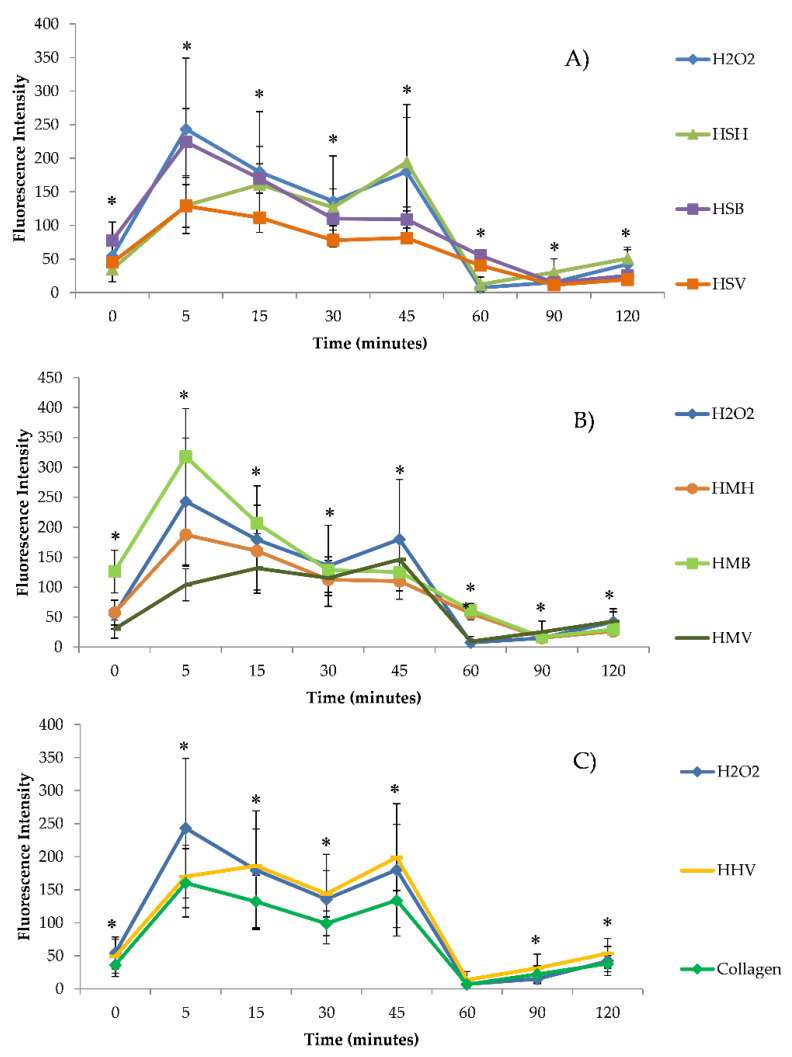
Time dependence of ROS-induced fluorescence in Caco-2/TC7 cells exposed to (**A**) salmon hydrolysates, (**B**) mackerel hydrolysates, and (**C**) herring hydrolysates and fish collagen at 1 mg/mL with oxidative stress induced by H_2_O_2_. Results are expressed as the mean ± SEM (*n* = 3). (*) *p* *≤* 0.05 indicates significant differences compared to the H_2_O_2_.

The intracellular accumulation of ROS in Caco-2/TC7 cells exposed to hydrolysates (HSV, HSB, HSH, HMH, HMB, HMV, HHV, and collagen) (1 mg/mL) without (Figure 3) and with (Figure 4) H_2_O_2_ was analyzed using H_2_-DCFDA. Figure 3 shows ROS generation grouped by type of fish hydrolysate. Figure 3 shows differences between ROS generation of hydrolysates with respect to the control. In general, ROS production increased slightly at 5 min, and then decreased and stabilized 60 min after exposure, at lower levels than at trial start, but without significant differences with respect to the control. Only significant differences of HMH and collagen, at 90 and 15 min, respectively, were determined with respect to the control (Figure 3; Table 1). The production of ROS in Caco-2/TC7 cells treated with salmon heads hydrolysate (HSH) and collagen was lower, whereas was higher in those treated with HSB, HSV, HMH, and HMB as compared to the control (Table 1). No differences were found between HMV, HHV, and control (Table 1). Among the different hydrolysates, the lowest ROS accumulation in Caco-2/TC7 cells was observed by exposure with HSH, followed and at increasing ROS accumulation levels by collagen, then HMV, HHV, HMB, HSB, HMH, and lastly HSV at 120 min, but not all differences were statistically significant (Table 1). Significant differences appeared at 15 min exposure, when higher ROS accumulation was found in the HMH and HSV as compared to the HSH treatment (*p =* 0.003; Tukey HSD), and again at 90 min exposure, when ROS accumulation was significantly higher in HSB as compared to the collagen treatment (*p =* 0.001; Tukey HSD).

Figure 4 shows ROS generation of hydrolysates exposed simultaneously to H_2_O_2_ grouped by type of fish hydrolysate. The ROS generation of hydrolysates with H_2_O_2_ was compared with H_2_O_2_ tested alone. When Caco-2/TC7 cells were exposed to oxidative stress induced by H_2_O_2_, they showed an increase in ROS levels which ranged from 1.9-fold to 17.2-fold at 0 and 45 min, respectively, compared to the control. Nevertheless, at 5 min collagen and most fish hydrolysates except HMB induced lower ROS accumulation as compared to H_2_O_2_ treatment alone, statistically significant only for HSH, HSV, and HMV (*p =* 0.000; Tukey HSD). After 5 min, the strongest antioxidant effect was induced by HMV. The antioxidant effect of fish hydrolysates was maintained until 45 min following exposure, and gradually disappeared as compared to the H_2_O_2_ treatment at 60 min following exposure, when there were higher ROS levels analyzed in HSV, HSB, HMH, and HMB treatments as compared to both the control and the H_2_O_2_ treatment (Figure 4). Considering the whole trial period, it was collagen which induced significant antioxidant effects against H_2_O_2_ exposure, and HHV which induced the highest ROS accumulation, though not significantly as compared to all treatments (Table 2).

### 3.3. Lipid Peroxidation Assay

**Figure 5:**
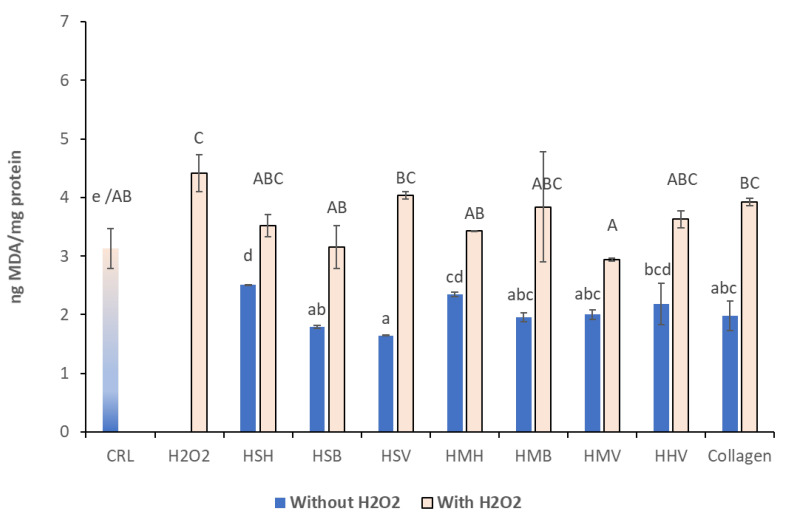
The LPO measured by MDA production in Caco-2/TC7 cells incubated with hydrolysates HMH, HSV, HSB, HMB, HHV, HSH, HMV, and collagen (1 mg/mL) with and without oxidative stress (H_2_O_2_). Results are expressed as the mean ± SEM (*n* = 3) in ng of MDA/mg of protein measured by the Lowry method. Values with common small or capital letters, for the treatment without or with H_2_O_2_ induced stress, respectively, are not significantly different (*p <* 0.05; Tukey HSD). CRL: control.

The LPO on Caco-2/TC7 cells was determined by the TBARS method in the presence of hydrolysates HSV, HSB, HSH, HMH, HMB, HMV, HHV, and collagen (1 mg/mL) with and without H_2_O_2_. As shown in Figure 5, the incubation for 24 h in the absence of oxidative stress significantly decreased the LPO production in Caco-2/TC7 cells (*p* *≤* 0.05). Cells treated with H_2_O_2_ alone showed significant LPO increase compared to the control, whereas all the hydrolysate treated cells showed similar LPO levels to that of the control, though there was an overall increase in LPO in the combined hydrolysate/H_2_O_2_ treatments, compared to the non-pretreated cells, of a magnitude between 40.2 and 146.3%. Respect to hydrolysates simultaneously exposed to H_2_O_2_, HSB, HMH, and HMV showed cytotoxic protection respect to H_2_O_2_ exposure. The highest cytoprotective effect was obtained for HMV, significant only in comparison to HSV, collagen, and H_2_O_2_ treatments. In cells without H_2_O_2_ pretreatment, HSV offered highest antioxidant effect, and HSH the lowest.

### 3.4. Comparison of Cytotoxicity between pure Hydrolysates and Their Bioavailable Fraction

**Figure 6:**
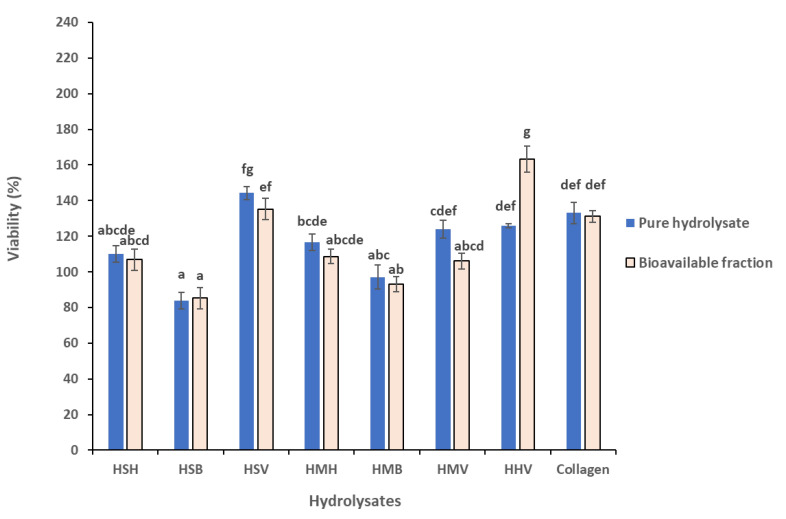
Viability (%) of the fish-based protein hydrolysates compared with the bioavailable fraction of the same protein hydrolysate at 1:16 dilution in Caco-2/TC7 cells after 24 h of exposure by MTT. Values are expressed as the mean ± SEM (*n* = 3). Values with common letters are not significantly different (*p <* 0.001; Tukey HSD).

In general terms, the bioavailable fraction of the tested fish hydrolysates showed similar effects as the respective pure hydrolysates. There were no significant differences in the cytoprotective effect of pure fish hydrolysates as compared to their bioavailable extracts (*p >* 0.1), with the exception of the bioavailable fraction of herring viscera hydrolysate (HHV) which increased cell viability significantly as compared to the respective pure hydrolysate (Figure 6) (*p <* 0.001; Tukey HSD).

Comparing the three salmon and three mackerel fraction hydrolysates together, the ANOVA analysis did not show significant species effects. On the other hand, significant differences were observed between the different side stream fractions. The backbone hydrolysate fractions did not induce a cytoprotective effect (−10.47% viability as compared to the pure hydrolysates). Head hydrolysates induced a 10.98% increase in cell viability, and the viscera induced significantly higher cell viability (28.78%) as compared to the other two fractions (HB^a^ < HH^b^ < HV^c^; *p <* 0.001; Tukey HSD). Comparing the visceral hydrolysate fraction effects, HMV (mackerel) induced significantly lower viability improvement (12.8% increased viability as compared to the pure hydrolysates) as compared to HSV (salmon) (39.72%), and HHV (herring) (50.70%) (*p <* 0.001; Tukey HSD).

### 3.5. Cytoprotective Effect of Hydrolysates against T-2 Mycotoxin

**Figure 7:**
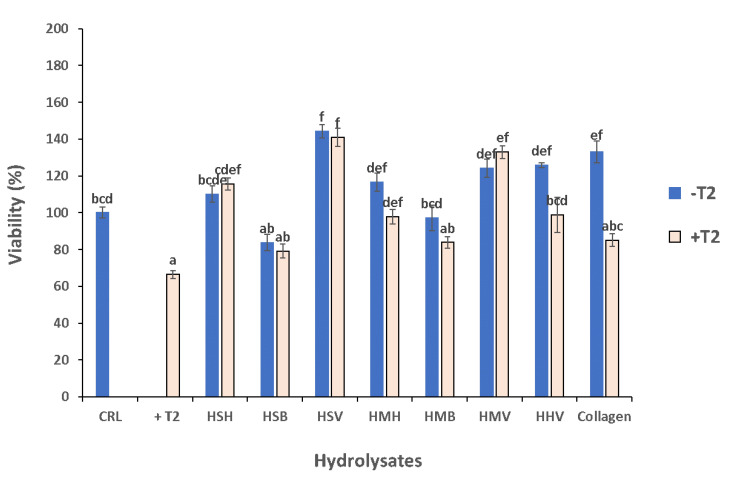
Viability (%) of T-2 toxin, pure hydrolysates, and hydrolysates combined with T-2 in Caco-2/TC7 cells after 24 h of exposure by MTT. All hydrolysates were added at 1:16 dilution and T-2 at 60 nM. Values are expressed as the mean ± SEM (*n* = 2). Values with common letters are not significantly different (*p <* 0.001; Tukey HSD). CRL: control.

Exposing Caco-2/TC7 cells to T-2 mycotoxin significantly reduced their viability. All tested fish side stream hydrolysates exerted cytoprotective effects against the T-2 mycotoxin, though not statistically significantly in the backbone hydrolysates HSB, HMB, and collagen as observed in Figure 7.

As seen in the viability trial without exposure to T-2 mycotoxin, the cytoprotective effect of salmon hydrolysates was higher than that of mackerel in cells exposed to T-2; this time the species factor showed a tendency for effect (*p =* 0.1). Likewise, the side stream fraction induced statistically significant effects, with the backbone fractions inducing the lowest cytoprotective effect (19.16% increased viability as compared to the T-2 treated cells), followed by the heads (43.46% increase in cell viability), and the viscera, which induced significantly higher cell viability (69.92%) as compared to the other two fractions (HB^a^ < HH^b^ < HV^c^; *p <* 0.001; Tukey HSD). Comparing the visceral hydrolysate fraction effects, it was HSV (salmon) which induced the highest cytoprotective effect against T-2 mycotoxin exposure (76.18% increased viability as compared to the T-2 treated cells), significantly higher as compared to HHV (herring) (62.47%), and HMV (mackerel) (60.80%) (*p <* 0.01; Tukey HSD).

### 3.6. Identification of Fungal Metabolites in Fish Hydrolysates

**Figure 8:**
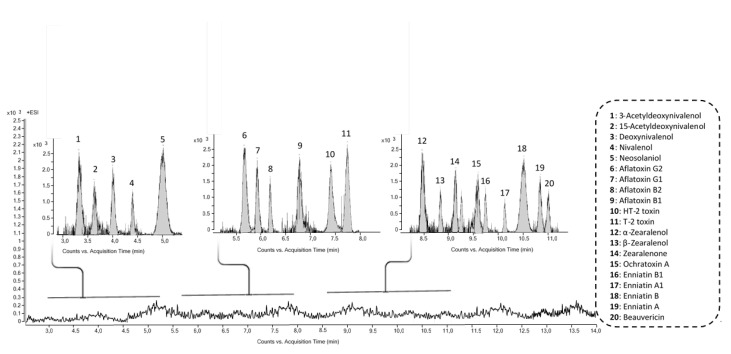
LC-Q-TOF MS extracted ion chromatogram of a pool sample of fish protein hydrolysates spiked with a mixture of mycotoxins.

The retrospective screening was applied to high resolution mass spectrometry data acquired in AutoMS/MS mode using a Q-TOF MS system. The screening of suspected fungal metabolites was performed using the MassHunter METLIN Metabolite PCD and PCDL. No fungal metabolites were identified in analyzed fish hydrolysates. To guarantee the quality control and quality assurance of the results, a pool sample of fish hydrolysates spiked with a mixture of mycotoxins at instrumental detection limit was performed and included in the same batch (Figure 8).

## 4. Discussion

Fish filleting side stream materials contain high levels of structural and bioactive proteins and smaller peptides, and lower levels of lipids containing long chain ω-3 polyunsaturated fatty acids (ω-3-PUFAs), phospholipids, bioactive nitrogenous compounds, organic minerals, and vitamins, among others. Most fish side streams are considered waste, though they are highly nutritious, containing, among other things, significant amounts of protein [49]. Effective use of fish side stream raw materials reduces the environmental impact of aquaculture and fisheries, and could provide high-added-value products to increase economical income for the marine product processing industries. In recent years, the use of extracts and compounds obtained from marine side stream biomass to improve the functionality of food products, in different processes such as organoleptic, health, and technological, has attracted much interest [50]. Among different applications, extracts and compounds obtained from fish side streams can be used as colorants, antimicrobials, antioxidant compounds, PUFAS, essential amino acids, and emulsifiers. These properties allow them to act as preservatives, to improve the nutritional and health profile of foods, as well as to improve the technological characteristics of the products. On the other hand, the use of these products may also have a great interest from the pharmaceutical point of view such as nutraceuticals, biorefinery, or development of new packaging systems [51].

In the present study, protein hydrolysates based on different side stream fractions from salmon, mackerel and herring, and flounder skin collagen have been selected to evaluate their potential use as functional food ingredients. Serial dilutions of each hydrolysate were chosen to study the potential cytotoxic/cytoprotective effects of the hydrolysates in Caco-2/TC7 cells. The hydrolysates did not show cytotoxic effects, however a cytoprotective effect was observed when Caco-2/TC7 cells where exposed to HSB at 0.125 mg/mL, and HSV at 0.0625 mg/mL and 0.25 mg/mL by MTT assay (Figure 1). The increase in cell viability at lower concentrations can be due to the hormetic effect. It can be observed as either a xenobiotic or an antioxidant substance, a hormone or a metabolite in several studies [52,53,54].

By the PC assay, all hydrolysates except HSB and HMV showed an increase in cell viability when Caco-2/TC7 cells were exposed to specific dilutions, including 0.0625 mg/mL (Figure 2). Wiriyaphan et al. [55] reported that hydrolysate of side streams of *Nemipterus* spp. did not have cytotoxic effects on Caco-2 cells. Furthermore, several studies on fish-based protein hydrolysates on multiple human cell lines have shown low toxicity and high cell viability [51,52]. Gómez et al. [56] showed a cytoprotective effect after 24 h of treatment with red tilapia side streams in Caco-2 cells, demonstrating an increase in cell viability in a dose-dependent manner. Similar to our results, in which the viscera hydrolysate showed highest cytoprotective effect, these authors demonstrated that the highest protection was achieved at 0.1 and 0.25 mg/mL for RTVH-A (red tilapia viscera hydrolysate with higher antioxidant activity) and FRTVH-V (red tilapia viscera hydrolysate with molecular weight cut-offs of <1 kDa fraction), respectively. In this line, Zhong et al. [57], found that protein hydrolysates isolated from silver carp by-products showed a cytoprotective effect on Caco-2 cells exposed to low concentrations of H_2_O_2_ and the highest capacity to neutralize radicals. Hu et al. [58] did not observe any significant cytotoxic effects on HepG2 cells after exposure of hydrolysate of monkfish (*Lophius litulon*) muscle during 24 h, by MTT assay. However, they reported an increase in a concentration-dependent manner of cell viability, compared with control, when the cells were exposed to peptides with antioxidant activity with H_2_O_2_-induced oxidative damage. For instance, the peptide MMP-12 increased the HepG2 cell viability from 48.85% ± 1.68% to 63.28% ± 2.06%, to 79.35% ± 2.85%, and to 88.65% ± 3.42% at the concentrations of 10, 50, and 100 µM, respectively. These findings suggest that the peptides from hydrolysate of monkfish muscle could strongly protect H_2_O_2_-induced oxidative damage HepG2 cells, especially at high concentrations. These results were consistent with the data presented by Zheng et al. [59], who showed that hydrolysate of swim bladders of *Nibea japonica* exposed to human umbilical vein endothelial (HUVECs) cells during 24 h did not decrease cell viability. Indeed, a collagen peptide of the swim bladders, named SNNH-1, promoted the growth of these cells. Moreover, Yang et al. [60] evidenced that marine collagen peptides from *Nibea japonica* skin have the potential to promote NIH-3T3 fibroblasts cells.

Reactive oxygen species (ROS) are generated in aerobic organisms during mitochondrial respiration to detoxify substances, chemical defense, cell signaling, and biosynthetic reactions [35,61]. When the elimination of ROS is inadequate, or their generation exceeds their elimination, ROS are accumulated in cells and oxidative stress occurs. Excess of ROS alters the normal cell metabolism oxidizing cell membrane phospholipids, proteins, lipids, DNA, and enzymes, among others [62]. Oxidative stress contributes to many noncommunicable disease pathologies such as neurodegenerative conditions, cardiovascular and inflammatory diseases, emphysema, and certain types of cancer [63]. Therefore, there is a significant interest in the development and application of functional food supplements with antioxidant properties to promote disease prevention and protect human health. The maintenance and proliferation of cell viability by a treatment with salmon, mackerel and herring hydrolysates could be related to the fact that these hydrolysates do not produce more ROS at a cellular level than cells not exposed to these hydrolysates. The ROS are generated by different physiological oxidative processes in the organisms, and associated with pathogenesis of various human diseases [64]. The overproduction of ROS culminates in oxidative damage to some key biological macromolecules which leads to significantly reduced cell viability [58]. In our study, none of the tested hydrolysates at 1 mg/mL concentration leads to a significant increase in the intracellular ROS levels in Caco-2 cells (*p >* 0.05) compared to the control, except HMH and collagen at 90 and 15 min, respectively (Figure 3; Table 1). However, cells pre-treated with H_2_O_2_ showed an increase in ROS generation at all times of exposure compared to the control (Figure 4). The decrease in intracellular ROS levels using a pretreatment with these hydrolysates suggested that sequestration or neutralization of free radicals is a possible mechanism of action in the antioxidant peptides found in hydrolysates. Similar results were obtained by Zhen et al. [59] who studied the effect of SNNH-1 on ROS levels in HUVECs cells. After H_2_O_2_ treatment, the fluorescence intensity in HUVECs cells was significantly higher compared to the control. However, SNNH-1 pretreatment reduced ROS levels in a concentration-dependent manner on HUVECs cells. These results were in agreement with the data presented by Li et al. [65], who found that collagen peptides from sea cucumbers (*Acaudina molpadioides*) could protect RAW264.7 cells from H_2_O_2_-induced damage. Hu et al. [58] observed the effects of MMP-4, MMP-7, and MMP-12 (three peptides of hydrolysate of monkfish, *Lophius litulon,* muscle), on the level of ROS in oxidative damage HepG2 cells. The level of ROS observed in HepG2 cells exposed to only H_2_O_2_ was 231.7% ± 13.5%, which was significantly higher than the control (100%). The intracellular ROS levels were significantly attenuated by MMP-4, MMP-7, and MMP-12 pretreatment in a dose-dependent manner. Among them, MMP-7 showed the strongest scavenging effect of ROS, and decreased the ROS level from 229.5% ± 16.8% to 165.2% ± 11.9%, to 137.3% ± 14.3%, and to 129.1% ± 8.6% at the concentrations of 10, 50, and 100 µM, respectively. Chen et al. [12] reported that the exposure of HepG2 cells to tilapia fish skin gelatin hydrolysates for 24 h decreased the level of ROS in a dose-dependent manner. Similarly, according to Wang et al. [32], the HepG2 cells caused significant inhibition of oxidative damage compared with cells treated only with H_2_O_2_, after pretreatment with mackerel proteins hydrolysate (5 mg/mL). Gómez et al. [56] determined that Caco-2 cells treated with H_2_O_2_ showed a 5 times increase in intracellular ROS levels compared to untreated cells, but these levels decreased by approximately 40% with pretreatment with red tilapia viscera hydrolysates RTVH-A and FRTVH-V. Other antioxidant peptides with high cellular antioxidant activity via neutralization of intracellular ROS have been identified in different sources of proteins, such as tilapia muscle [35] and tilapia scale gelatin [66].

Antioxidant collagen peptides are also known to provide cell protection from oxidative stress damage, through scavenging ROS and increasing the levels of intracellular antioxidant enzymes [67]. This work shows that flounder skin collagen protects cells from oxidative stress, decreasing ROS levels and MDA. Moreover, comparing with hydrolysates, it is amongst those which exert the most protective effect. These finding are corroborated with the results obtained by other authors. Collagen peptides from crimson snapper (*Lutianus erythroptrus*) scales [12] and royal jelly [64] could prolong the average life of Drosophila treated with H_2_O_2_ by decreasing the contents of peroxide products, such as MDA and protein carbonylation. Moreover, Wang et al. [68] determined that collagen of the red lip croaker could protect H_2_O_2_-damaged HepG2 cells from oxidative stress by decreasing ROS and MDA levels and enhancing endogenous antioxidant enzyme defense system, so it could serve as antioxidant used in food and health products. Moreover, collagen peptides are popular in cosmetics as, for example, they can protect the skin from ultraviolet (UV) radiation injuries [68].

The LPO occurs as a non-specific process secondary to initial cell damage, which can be blocked by antioxidants. The MDA is the oxidative metabolite of cell lipid oxidation and can attack unsaturated fatty acids in the cell membrane, which causes cell damage. Therefore, the degree of LPO and cell damage can be evaluated according to the MDA content in cells [30]. Our results showed that salmon, mackerel, and herring hydrolysates prevented the propagation of LPO, decreasing the MDA levels in Caco-2/TC7 cells. These results demonstrated that salmon (HSB) and mackerel (HMH and HMV) hydrolysates may protect Caco-2/TC7 cells from oxidative damage. Similar results were obtained by Zheng et al. [59], who reported that MDA content in HUVEC cells was significantly higher after H_2_O_2_ treatment compared to the control; however, pretreatment of HUVECs with SNNH-1 decreased the amount of MDA in a dose-dependent manner. In addition, Cai et al. [69] reported that FPYLRH (S8) from the swim bladders of miiuy croaker (*Miichthys miiuy*) could down-regulate the contents of MDA, suggesting that it plays a protective role in the antioxidant effects on HUVECs cells against H_2_O_2_-induced damage. Following this, Zhen et al. [59] determined the effect of SNNH-1 pretreatment on the levels of MDA after oxidative damage of HUVECs induced by H_2_O_2_. The results concluded that SNNH-1 inhibited intracellular LPO and enhanced the cell´s antioxidant defense system. Hu et al. [58] studied the effects of protein hydrolysate of monkfish on MDA levels in H_2_O_2_-induced HepG2 cells. The MDA content (21.63 ± 0.81 nM/mg protein) in HepG2 cells exposed to H_2_O_2_ was significantly increased compared with the control group (9.32 ± 0.35 nM/mg protein). At the 10, 50, and 100 µM concentration, the MDA content of MMP-12 peptide was 18.8 ± 0.56, 16.46 ± 0.74, and 12.43 ± 0.62 nM/mg protein, respectively, which was lower than those of MMP-4 and MMP-7 peptides, and the H_2_O_2_ treated group. Therefore, MMP-4, MMP-7, and MMP-12 peptides could reduce the oxidative stress injury by the decrease in LPO.

The results obtained in this study indicate that fish protein hydrolysates represent great candidate molecules for the development of antioxidant dietary supplements. However, peptides are often chemically and physically unstable, display rapid clearance, and are quickly degraded by enzymes [70]. Therefore, ensuring the bioaccessibility of peptides should be evaluated. Thus, the bioavailable fraction of the different protein hydrolysates was achieved and a comparison between the cell viability of pure hydrolysates and their bioavailable fraction was carried out. These results revealed the same trend as the as the pure hydrolysates. This indicates that there were no statistically significant differences, in terms of toxicity or antioxidant capability, of the protein hydrolysates once the digestion process had been carried out.

Additionally, the viability of cells exposed to pure hydrolysates and to hydrolysates combined with T-2 was compared to corroborate the cytoprotective effect of fish hydrolysates. The results evidenced that almost all the hydrolysates combined with T-2 showed an increase in viability compared to cell exposure to T-2 alone. This may be due to the fact that these hydrolysates counteract the cytotoxic effect of T-2, and they act as if the cell were only exposed to the hydrolysate. Hydrolysates based on viscera, regardless of the species, combined with T-2, exercised the same effect on cell viability than if they were not exposed to T-2, therefore viscera hydrolysates exert the most protective effect. As can be observed in Figure 7 and according to MTT assay (Figure 1), 1:16 dilution of HSV exercised the most cytoprotective effect compared with the rest of hydrolysates. Moreover, when hydrolysates were combined with T-2, HSV also exercised the most cytoprotective effect. However, when collagen was combined with T-2, we saw significantly decreased cell viability with respect to its corresponding pure hydrolysates. This could be as collagen evidenced the highest bacterial content of all hydrolysates analyzed (data not shown), which may be related with conditions of the degradation, or breakdown, of the fish. Furthermore, collagen evidenced higher levels of trimethylamine-N (TMA; 66 mg N/100 g) [42], which is an indicator of poor product quality. This could explain the fact that a co-exposure of collagen with T-2 decreased the viability, as the collagen did not have the necessary quality parameters to protect the cell against this damage.

Finally, it is important to know whether the feedstuffs used for farmed fish are contaminated. Fish from aquaculture are fed using different feeds and raw materials from vegetal origin and plant products, which could be contaminated by mycotoxins [71]. Different studies have shown that some mycotoxins carry-over from feed into edible tissues or fluids from animals [72,73,74]. As far as the foodstuffs from aquaculture industries are concerned, the occurrence of aflatoxins, ochratoxin A, and *Fusarium* mycotoxins, such as thrichotecenes and the emerging fusariotoxins, have been reported in the literature [75,76,77,78,79]. Tolosa et al. [76] determined the occurrence of enniatins (ENs) in feed for farmed fish and in fish samples of sea bass and sea bream. They reported an occurrence of ENs in up to 25% of analyzed samples (*n* = 20) at levels from 1.01 to 119.0 µg/Kg, and revealed higher average concentrations of EN A1 (11.1 µg/Kg), EN B (13.0 µg/Kg), and EN B1 (19.0 µg/Kg) in head than in liver (5.1, 12.6, and 5.3 µg/Kg for EN A1, EN B, and EN B1, respectively). Those findings corroborate the ability of ENs to distribute and persist into tissues, and even penetrate different barriers, including the blood-brain barrier, in higher organisms as previously evidenced in the literature [80,81]. However, mycotoxins were not detected in the protein hydrolysates, suggesting that the fish had not been exposed to mycotoxins. 

To summarize, HSV showed the highest cytoprotective effect in Caco-2/TC7 as evidenced by MTT, ROS, and LPO assays. HMV treatment also showed a reduction in ROS values when compared to H_2_O_2_. On the other hand, the HHV bioavailable fraction showed the highest percentage of cell viability in Caco-2/TC7 cells compared to the rest of hydrolysates. Moreover, HSV presented the highest cytoprotective effect when hydrolysates are combined with T-2 toxin.

## 5. Conclusions

Taken together, the findings from this study suggest that heads, backbones, and viscera from salmon, mackerel, and herring could be utilized as valuable raw materials in the development of health-promoting functional foods with potential antioxidant capacities. Both hydrolysates and their bioavailable fraction induced similar cell viability rates. Moreover, from the results obtained, it can be concluded that the highest cytoprotective effect was obtained for the salmon viscera protein hydrolysate. In addition, ROS accumulation induced by H_2_O_2_ and LPO was suppressed by all pure hydrolysates, and the cytoprotective effect of all hydrolysates was observed against T-2. This scientific basis may provide a solution for dealing with the high cost and environmental problems associated with the disposal of such waste material. However, more studies are needed to investigate the biological effects of fish hydrolysates in order to transform protein rich side stream products into valuable products, as well as to ensure food safety.

## Figures and Tables

**Table 1:** Outcomes of the differences between hydrolysates and collagen without H_2_O_2_ and control from 0 to 120 min of exposure using ANOVA followed by a Tukey HDS post-hoc test for multiple comparisons.

			Salmon			Mackerel			Herring
		CTR	HSH	HSB	HSV	HMH	HMB	HMV	HHV
Salmon	HSH	HSH < CTR (0.1 > *p* > 0.05)							
	HSB	HSB > CTR (0.1 > *p* > 0.05)	HSB > HSH (*p* ≤ 0.05)						
	HSV	HSV > CTR (0.1 > *p* > 0.05)	HSV > HSH (*p* ≤ 0.05)	NS					
Mackerel	HMH	HMH > CTR (*p* ≤ 0.05)	HMH > HSH (*p* ≤ 0.05)	NS	NS				
	HMB	HMB > CTR (0.1 > *p* > 0.05)	HMB > HSH (0.1> *p* > 0.05)	NS	NS	NS			
	HMV	NS	NS	HMV < HSB (*p* ≤ 0.05)	HMV < HSV (*p* ≤ 0.05)	HMV < HMH (*p* ≤ 0.05)	HMV < HMB (*p* ≤ 0.05)		
Herring	HHV	NS	HHV > HSH (0.1 > *p* > 0.05)	HHV < HSB (0.1 > *p* > 0.05)	HHV < HSV (0.1 > *p* > 0.05)	HHV < HMH (*p* ≤ 0.05)	HHV < HMB (0.1 > *p* > 0.05)	HMV < HHV (0.1 > *p* > 0.05)	
Flownder	Collagen	Collagen < CTR (*p* ≤ 0.05)	NS	Collagen < HSB (*p* ≤ 0.05)	Collagen < HSV (*p* ≤ 0.05)	Collagen < HMH (*p* ≤ 0.05)	Collagen < HMB (*p* ≤ 0.05)	Collagen < HMV (*p* ≤ 0.05)	Collagen < HHV (*p* ≤ 0.05)

NS: non-significant.

**Table 2:** Outcomes of the differences between hydrolysates and collagen with H_2_O_2_ and control from 0 to 120 min of exposure using ANOVA followed by a Tukey HDS post-hoc test for multiple comparisons.

				Salmon			Mackerel			Herring
		CTR	H_2_O_2_	HSH_H2O2_	HSB_H2O2_	HSV_H2O2_	HMH_H2O2_	HMB_H2O2_	HMV_H2O2_	HHV_H2O2_
	H_2_O_2_	H_2_O_2_ > CTR (*p* ≤ 0.05)								
Salmon	HSH_H2O2_	HSH > CTR (*p* ≤ 0.05)	NS							
	HSB_H2O2_	HSB > CTR (*p* ≤ 0.05)	NS	NS						
	HSV_H2O2_	HSV > CTR (*p* ≤ 0.05)	HSV < H_2_O_2_ (0.1> *p* > 0.05)	NS	HSV < HSB (*p* ≤ 0.05)					
Mackerel	HMH_H2O2_	HMH > CTR (*p* ≤ 0.05)	NS	NS	NS	HMH > HSV (*p* ≤ 0.05)				
	HMB_H2O2_	HMB > CTR (*p* ≤ 0.05)	NS	NS	HMB > HSB (*p* ≤ 0.05)	HMB > HSV (*p* ≤ 0.05)	HMB > HMH (0.1> *p* > 0.05)			
	HMV_H2O2_	HMV > CTR (*p* ≤ 0.05)	NS	HMV < HSH (*p* ≤ 0.05)	NS	NS	NS	NS		
Herring	HHV_H2O2_	HHV > CTR (*p* ≤ 0.05)	NS	HHV > HSH (*p* ≤ 0.05)	NS	HHV > HSV (*p* ≤ 0.05)	NS	NS	HHV > HMV (*p* ≤ 0.05)	
Flownder	Collagen_H2O2_	Collaben > CTR (*p* ≤ 0.05)	Collagen < H_2_O_2_ (*p* ≤ 0.05)	NS	Collagen < HSB (*p* ≤ 0.05)	NS	Collagen > HMH (0.1> *p* > 0.05)	Collagen < HMB (*p* ≤ 0.05)	NS	Collagen < HHV (*p* ≤ 0.05)

NS: non-significant.

## Data Availability

Data is contained within the article.
